# Impact of Residual Fragments following Endourological Treatments in Renal Stones

**DOI:** 10.1155/2012/813523

**Published:** 2012-07-05

**Authors:** Cenk Acar, Cag Cal

**Affiliations:** ^1^Department of Urology, Faculty of Medicine, Pamukkale University, 20070 Denizli, Turkey; ^2^Department of Urology, Faculty of Medicine, Ege University, 35100 Izmir, Turkey

## Abstract

Today, shock wave lithotripsy (SWL), percutaneous nephrolithotomy (PCNL), and flexible ureterorenoscopy (URS) are the most widely used modalities for the management of renal stones. In earlier series, treatment success of renal calculi assessed with KUB radiography, ultrasound, or intravenous pyelography which are less sensitive than CT that leads to be diversity of study results in reporting outcome. Residual fragments (RFs) after interventional therapies may cause pain, infection, or obstruction. The size and location of RFs following SWL and PCNL are the major predictors for clinical significant symptoms and stone events requiring intervention. There is no consensus regarding schedule for followup of SWL, PCNL, and flexible URS. Active monitoring can be recommended when the stones become symptomatic, increase in size, or need intervention. RFs <4 mm after SWL and <2 mm after PCNL and flexible URS could be actively monitored on an annual basis with CT. Early repeat SWL and second-look endoscopy are recommended after primary SWL and PCNL, respectively. There is insufficient data for flexible URS, but RFs can be easily treated with repeat URS. Finally, medical therapy should be tailored based on the stone analysis and metabolic workup that may be helpful to prevent regrowth of the RFs.

## 1. Introduction

Urolithiasis is a common disorder, affecting 3% to 5% of the population in industrialized countries and gradually increasing all around the world [1, 2]. Today, shock wave lithotripsy (SWL), percutaneous nephrolithotomy (PCNL), and flexible ureteroscopy (URS) are widely used for renal stones. The overall success rates of SWL, PCNL, and URS in renal stones are 13.6–91.2% [3–5], 40–90% [6], and 34–92.2% [7, 8] in different studies, respectively. The success of these treatments also accepted free-of-stone rates in a radiologic studies such as kidney ureter bladder (KUB) radiography, ultrasonography (US), and abdominal computerized tomography (CT). CT is certainly the most sensitive method for assessing residual stones. Although CT detects stone fragments down to 1 mm in all subtypes of stones (radiolucent uric acid or cystine calculi as well as calcium-containing stones), the clinical value of being able to detect very small fragments remains debatable [9]. Besides, detecting negligible RF with CT is time-consuming and expensive. Although the renal stone protocol on newer multidetector scanners has been widely used, they lead to expose 25–50-fold higher doses of radiation than KUB radiography [10]. The advantages of ultrasound are providing comparable precision and detecting stones down to a diameter of 2 mm [11]. The clinicians suggested that evaluation of asymptomatic patients after SWL could be limited to routine radiologic studies such as KUB radiography and US [11–14].

Clinically insignificant residual stone fragments (CIRF) defined as residual calculi <4 or 5 mm while the patient is asymptomatic and the stone composition is not struvite or infection stone [15–17]. Residual fragments (RF) may cause pain, infection, or obstruction whether clinically significant or not. These patients could need auxiliary measures, including further SWL sessions, double-J stent insertions, or endoscopic surgical treatment. In addition, RFs are important that may act as nidus for regrowth. At this point of view, term of CIRF reflects a common and still controversial problem for SWL, PCNL, or URS.

To define follow-up protocols and timing of auxiliary intervention, better understanding of the natural history of RFs following endourological treatments in renal stones should be needed. In this paper, we discussed fate of RF following SWL, PCNL, and flexible URS in kidney stones with recent literature.

## 2. Residual Fragments following SWL

In recent literature, several studies evaluated the fate of RFs after SWL [11–14, 18]. Spontaneous stone passage was found in 13.6% to 51.3% of the patients with mean followup between 15 and 57 months. Stone size remained stable in 17.3% to 52.6% of the patients, while stone regrowth was encountered in 21.4% to 69%. El-Nahas et al. reported that 52 (33.7%) of 154 patients had significant clinical stone event that need further intervention (mostly SWL) [18]. Osman et al. noted that 21.4% of the residual stone led to stone recurrence and need for retreatment [14]. The spontaneous clearance rate was affected by location of RF. The highest rates are seen in the ureter. In addition, many investigators emphasized that RFs are commonly localized to lower-pole calyces after SWL wherever treated in the kidney [5]. In a review, the authors concluded that 25% of the patients with CIRF will become stone-free, 50% will remain symptom-free, 20% will experience a clinically significant stone episode, and 4% to 25% of patients will need a secondary intervention, especially SWL [16]. According to this prediction, auxiliary endoscopic intervention should be considered as overtreatment in asymptomatic patients [16]. In a recent systematic review, the natural history and clinical significance of small and asymptomatic RFs after SWL were extensively discussed with data of four studies. Totally 463 patients with CIRF (<5 mm in diameter) were followed with a period of 15 months to 4.9 years. The stone-free rates were 23.8% to 78.9% and 10.7% to 41.9% of the stones remained stable. The stone size increased in 2% to 58.6% of the patients. Between 41.4% and 100% of the patients were asymptomatic while up to 58.6% had a symptomatic episode or needed intervention with a mean followup at 15 to 57 months after SWL [19].

Most investigators indicated that the risk factors of CIRF becoming clinically significant are stone burden and number and location of RF [14, 18]. The rate of complications and number of auxiliary interventions increased during long follow-up period [11]. El-Nahas et al. noted that patients with previous stone disease had 4.26 times higher incidence for stone events [18]. In a prospective study, the authors could not find any relationship between metaphylaxis and stone regrowth [14]. The results of these studies reflect that significant numbers of patients will need auxiliary intervention or be symptomatic during followup of SWL treatment. Consequently, the patients with RF should be actively monitored whether clinically significant or not.

On the other hand, in a prospective randomized comparative study, the author suggested that SWL retreatment promotes discharge of persistent caliceal RF after primary SWL [20]. However, it is taking into consideration the potentially higher side effects and additional cost against the advantages of a higher stone-free rate by SWL retreatment.

As a conclusion, there is no consensus regarding schedule for followup. Active monitoring can be recommended when the stones become symptomatic, increase in size, or need intervention. Most studies reported that the clearance rate is higher at 6 months, but spontaneous clearance may last up to 24 months [11, 16, 18]. In standard followup, the authors recommended clinical examination, urine culture, and routine radiographic studies as KUB radiography and/or renal US at 3-, 6-, or 12-month intervals. Such an intense followup may not be indicated for all patients because spontaneous stone clearance asymptomatically occurs in most of the patients. Therefore, any auxiliary procedures are rarely required. We recommend the initial followup can be performed at 6 months and it can be followed by an annual schedule for the patients who were not clear at that time. The intervention should be relatively noninvasive and consisted of either repeated SWL or retrograde endoscopy.

## 3. Residual Fragments following PCNL

Percutaneous nephrolithotomy is an effective minimally invasive procedure that is preformed for treatment of large and complex calculi [21]. Altunrende et al. investigated the medium-term outcome of CIRFs. They also measured 24-hour urine metabolic analysis. After PCNL, 38 patients with CIRFs followed periodically in a median time of 28.4 months. Ten (26.3%) patients had symptomatic episode that need medical therapy. The RF size was stable or decreased in 27 (71.1%) cases whilst increased in 8 (21.1%) patients. Spontaneous stone rate was 7.9% in medium term of followup. They concluded that the progression could be seen in 2-year followup, whereas presence of risk factors on 24-hour urine metabolic analysis could not predict growth of RFs [22]. Ganpule and Desai retrospectively examined in 187 patients to evaluate the fate of residual stones after PCNL [23]. 57.7% of RF was located in the lower calix, and the mean size of RF was 38.6 ± 52 mm^2^. Fourty-five percent of the patients were stone-free without intervention at a mean followup of 24 months. Most of the stones (65.47%) spontaneously passed in 3 months. Small RFs (<25 mm^2^) in renal pelvis had best clearance rate. In addition, a history of previous intervention, renal failure, metabolic abnormalities such as hyperuraecemia and hypercalciuria, size of RF, and experience of surgeon affected the fate of RF after PCNL in multivariate analysis. Raman et al. prospectively evaluated 42 patients with RF after PCNL with a median followup of 41 months [24]. They performed abdominal CT to determine the RF and classify the RF by using cut-off size as 2 mm. Sixty percent of RF was 2 mm or smaller and 79% was <5 mm. The location of RF was 47% lower, 32% middle, 24% upper pole, and 18% renal pelvis/ureter. Forty-three percent of patients experienced a stone-related event with a median time to occurrence at 32 months. Of these patients, 61% underwent auxiliary procedures such as URS, PCNL, SWL, nephrectomy, and double-J stent placement, but mostly URS. They calculated the estimated 3- and 5-year event-free probabilities were 74% and 48%, respectively. On multivariate analysis, a maximum RF size >2 mm and location in the renal pelvis or ureter independently predicted a stone-related event. The probability of needing a second surgical procedure correlated with RF size. Eight percent of patients with RFs <2 mm underwent surgical retreatment compared with 53% of patients with RFs >2 mm. These results indicate that patients with RFs >2 mm or fragments located in the renal pelvis or ureter should be treated immediately. On the other hand, some authors evaluated cost comparison of immediate second-look endoscopy against surveillance in post-PCNL RFs. They concluded that second-look endoscopy is cost advantageous in the patients with RF >4 mm following PCNL which generally leads to stone event [25].

The schedule of followup following PCNL is still controversial. Taking into consideration that the majority of these stones will pass spontaneously without causing any symptoms during the first year of followup, an annual followup with a CT scan could be sufficient [19].

## 4. Residual Fragments following Flexible URS

Owing to technological advances, flexible URS has expanded its indications to include the stones in kidney [26]. The indication of flexible URS for renal stones is the stones less than 15 mm which do not respond to SWL [27]. The one of the advantages of flexible URS is the possibility of treating renal and ureteral stones in the same patient in a single session. In early series, treatment success of renal calculi was assessed with KUB radiography, US, or intravenous pyelography which are less sensitive than CT that leads to be diversity of study results. Breda et al. evaluated 51 patients with 161 renal stones (mean stone size of 6.6 mm) and they found the overall stone-free rate after single and second procedures was 64.7% and 92.2%, respectively [8]. In a different study, Perlmutter et al. evaluated the impact of stone location on success rates of flexible URS [28]. A total of 86 renal stones were treated, and the stone-free rates for upper-, middle-, and lower-caliceal stones were 100%, 95.8%, and 90.9%, respectively. They concluded that stone location does not significantly affect stone clearance rates. In a prospective randomized trial, Pearle et al. compared SWL (32 patients) and URS (35 patients) for lower-pole caliceal stones of 1 cm or less [29]. The stone-free rates of SWL and flexible URS were 35% and 50%, respectively. They concluded that there was a statistically insignificant difference in stone-free rates between SWL and URS for the treatment of small lower-pole renal calculi. However, SWL was associated with greater patient acceptance and shorter convalescence. Tanriverdi et al. evaluated the stone-free rate of flexible URS in renal calculi by classifying the RFs as the largest single fragment <2 mm, <4 mm, and >4 mm in CT [30]. The success rates were 50.4%, 62.8%, and 84.1% in stone-free, CIRF <2 mm and CIRF <4 mm, respectively.

According to aforementioned studies, all RFs may be regrowth and become symptomatic regardless of stone size [13, 24, 30]. In a recent study, Rebuck et al. evaluated the fate of RFs following flexible URS in 51 patients with renal calculi [31]. Although the follow-up protocol was complex, patients with RFs actively monitored with abdominal CT and term of CIRF (≤4 mm) was used for RFs. They excluded patients who had fragments in ureter after URS and patients with complex renal anatomy. Mean follow-up duration was 18.9 months. During the follow-up period, 19.6% of the patients experienced at least one stone event, 21.7% spontaneously passed their fragments, and 58.7% remained asymptomatic over time. The stone event occurs in mean followup of 26.8 months. Most of the patients with stone event treated with repeated URS. Among the patients with spontaneous passage, 60% passed their fragments after a mean followup of 9.1 months. In contrast to several studies, stone regrowth rate was very low in this study. In addition, the authors noted that stone events occur rather than lower-pole RF location while most of remaining stones located in lower pole. They attributed these results to small sample size, compliance of their patients against stone prevention therapy, and short followup of patients with asymptomatic RFs. As a conclusion, approximately one in five patients will experience stone events over 1.6 years after flexible URS and most of them can easily be treated with URS. Although, there is no consensus on the schedule of followup, asymptomatic patients with RF can actively monitor with abdominal CT until patients become symptomatic or 2 years of followup.

## 5. Medical Management on RF following Endourological Procedures

Several authors discussed medical therapy in the management of RFs after SWL or PCNL [32–36]. Fine et al. evaluated 80 patients to determine the effect of medical therapy in a retrospective study. Patients who received medical therapy had significant decrease in the stone-formation rate from a median of 1.17 to 0.00 stones per patient per year while the patients who did not receive medical therapy had a minimal decrease from a median of 1.33 to 0.77 stones per patient per year. The medically treated patients had a significantly greater stone remission rate (63.9% versus 23.1%) and lower stone burden increase (27.8% versus 61.6%) than the untreated patients. Regarding patients with CIRF, 16% of medically treated patients had fragment regrowth compared with 54.5% of the untreated patients. The authors concluded that medical therapy may alleviate stone regrowth in patients with residual stone fragments after SWL [33]. Cicerello et al. evaluated the effect of alkaline citrate therapy in patients with RF <5 mm [32]. In up to 74% of calcium oxalate patients and in up to 86% of infection stone patients, the RFs became undetectable in 1 year of followup. The undetectable RF rates of the patients were 32% and 40% in the control group for calcium oxalate and infection stones, respectively. RF regrowth rate decreased 47% to 5% in calcium oxalate patients with medical treatment. No significant difference was found for infection stones [32]. Similarly, Soygür et al. reported that among the patients with lower-pole RFs after SWL, the patients who were receiving citrate therapy showed a significantly higher stone fragment-disappearance rate (45.5% versus 12.5%) and less stone recurrence rate (56.6% versus 87.5%) compared with the control group at 12 months of followup [36]. Kang et al. evaluated the effect of medical therapy management in patients with RFs following PCNL. Patients receiving medical therapy had a lower median stone-free rate (0.02 versus 1.00 stones per patient per year) and a higher remission rate (77% versus 21%) compared with patients not receiving medical therapy. This result indicated that medical therapy can inhibit new stone formation or growth in patients with residual fragments after PCNL [34]. Afterwards, there is little evidence related to effectiveness of medical therapy in patients with RFs after flexible URS. Rebuck et al. speculated the high rate of stones remaining stable was related to medical therapy strategy in particularly lower-pole location [31].

There is no valuable data for determining the cost effectiveness of medical therapy after interventional therapies. However, the cost analysis of interventional therapies may help to make better estimation for medical management. Few studies have evaluated the cost of different treatment strategies for treatment of renal calculi. Koo et al. compared the cost effectiveness and outcome of SWL and flexible URS for lower-pole renal calculi ≤20 mm [37]. The costs of flexible URS and SWL for each session were calculated approximately $4,116 and $673, respectively. In another study, comparing clinical outcomes and the estimated cost of PCNL and flexible URS for 2 to 3 cm renal stones, the estimated cost of PCNL was significantly greater than flexible URS ($19,845 versus $6,675, resp.) [38]. Regarding to determine the cost effectiveness of medical therapy and prophylaxis, Lotan et al. published an international comparison for costs of medical treatments [39]. The authors classified the treatments into 3 categories as conservative therapy (comprised dietary modification without drug treatment or metabolic evaluation), empiric medical therapy (both drug and dietary treatment were initiated without metabolic evaluation in all patients), and directed medical therapy (based on comprehensive metabolic evaluation). The study result showed that conservative therapy was the least costly strategy in all countries except the UK, but was associated with the highest stone recurrence rate (0.3 stones/patient/year) for recurrent stone formers. The empiric therapy amounted $508 in America, $150 in Turkey, and $29 in UK per patient in one year. In other countries with relatively low-medication costs, only small differences between empiric and conservative therapy were demonstrated. Drug therapy (empiric and directed) was associated with a significantly lower rate of stone recurrence than conservative therapy. The authors emphasized that empiric therapy was minimally more effective than directed therapy because of effectiveness of medication in all treated patients whether they had a metabolic abnormality or not. Thus, medical treatment after interventional therapy seems to be more cost effective than auxiliary treatments for RFs. As a conclusion, medical therapy after endourological treatment should not replace the appropriate interventional therapy that the best chance to achieve a stone-free status with minimal morbidity. However, metabolic workup should immediately be performed before stone therapy. It may help to diagnose underlying metabolic abnormality and medical therapy may be begun promptly.

## 6. Summary

The size and location of RFs following SWL and PCNL therapies are the major predictors for clinical significant symptoms and stone events which requiring intervention. RFs <4 mm after SWL and <2 mm after PCNL and flexible URS could be actively monitored on an annual basis with CT. Early repeat SWL and second-look endoscopy are recommended after primary SWL and PCNL, respectively. There is no sufficient data for flexible URS but RFs can be easily treated with repeat URS. Finally, medical therapy according stone analysis and metabolic workup might be helpful to prevent regrowth of the RFs.
